# OneStop: A 360-Participant English Eye Tracking Dataset with Different Reading Regimes

**DOI:** 10.1038/s41597-025-06272-2

**Published:** 2025-12-03

**Authors:** Yevgeni Berzak, Jonathan Malmaud, Omer Shubi, Yoav Meiri, Ella Lion, Roger Levy

**Affiliations:** 1 Technion - Israel Institute of Technology, Faculty of Data and Decision Sciences, Haifa, Israel; 2 Google DeepMind, Mountain View, USA; 3 Massachusetts Institute of Technology, Department of Brain and Cognitive Sciences, Cambridge, USA

**Keywords:** Human behaviour, Human behaviour

## Abstract

We present OneStop Eye Movements, a large-scale corpus of eye movements in reading, in which native (L1) speakers read newswire texts in English and answer reading comprehension questions. OneStop has 152 hours of eye movement recordings from 360 participants for 2.6 million word tokens, more data than all the existing public broad coverage English L1 eye tracking datasets combined. The eye movement data was collected for extensively piloted reading comprehension materials comprising 486 reading comprehension questions and auxiliary text annotations geared towards behavioral analyses of reading comprehension. Importantly, OneStop includes multiple reading regimes: ordinary reading, information seeking, repeated reading of the same text, and reading simplified text. The combination of the unprecedented size, high-quality reading comprehension materials and multiple reading scenarios, aims to enable new research avenues in the study of reading and human language processing. It further aims to facilitate the integration of eye tracking data in Natural Language Processing (NLP), Artificial Intelligence (AI), Human Computer Interaction (HCI) and educational applications.

## Background & Summary

Eye tracking is one of the most important and widely used methodologies in the study of reading and human language processing. Over the past few decades, considerable efforts have been invested in the collection of eye tracking for reading data, in particular broad coverage data during reading of free-form text^1–8^. Such datasets have proved to be highly valuable for testing and generating cognitive theories of human language processing, and for integrating psycholinguistics with NLP, AI, and adjacent fields^9–15^.

However, despite the clear utility of existing datasets, the potential of this type of data is far from being fully seized. Broad coverage eye tracking datasets for reading are still few and limited in size, and the amount of existing data is not sufficient for many potential research purposes, especially those involving individual differences across readers and the utilization of machine learning and AI tools. Further, although one of the central research avenues in psycholinguistics concerns the relations between eye movements and language comprehension, eye tracking for reading experiments often neglect rigorous behavioral testing of reading comprehension. Publicly available eye tracking datasets either completely forgo testing for reading comprehension or use simple ad-hoc questions that serve primarily as attention checks. Such questions cannot provide a solid foundation for studying high-level reading comprehension. To our knowledge, SB-SAT^6^ is currently the only publicly available eye tracking dataset for English which includes a high-quality reading comprehension component. Finally, existing broad coverage eye tracking datasets focus on a single reading regime - reading an arbitrary text for the first time, without a specific goal. Despite its ubiquity in reading experiments, this scenario covers only a fraction of our daily life reading experience, leaving many common reading regimes understudied.

The primary goal of OneStop^16^ is to enable new types of research on the relations between eye movements, reading comprehension, and different types of interactions of the reader with the text. This is facilitated through several key characteristics of the dataset. First is its size. OneStop is the largest eye tracking dataset in English L1 to date with respect to the number of participants, amount of collected eye tracking data and number of reading comprehension questions. It includes eye movement recordings from 360 English L1 participants for 2,632,159 word tokens, more data than all the existing eye tracking datasets for English L1 combined. OneStop further includes 486 reading comprehension questions, nearly 25 times more than SB-SAT.

OneStop has several additional characteristics that distinguish it from existing broad coverage eye tracking datasets for reading. OneStop is the first eye tracking dataset which includes a controlled manipulation of *text difficulty*, allowing to study the relations between text readability and eye movements. Its reading materials comprise 30 texts from the Guardian with diverse topics in two difficulty levels, the original Advanced level, and a simplified Elementary level. OneStop has 19,428 words in the Advanced version and 15,737 words in the Elementary version. Second, is the high quality and auxiliary annotations of the underlying reading comprehension materials. The OneStop materials are taken from the OneStopQA corpus^17^, and comprise extensively piloted reading comprehension questions with auxiliary text annotations that tie answers to their textual support. These annotations enable studying the relations between eye movement patterns over specific portions of the text and reading comprehension performance.

Different from other datasets where multiple questions are presented at once after reading a long text, in OneStop, reading comprehension is tested using a single question for a single paragraph immediately after paragraph reading. This design minimizes the effects of memory decay and interference from other questions. OneStop further decouples eye movements during text reading and question answering by presenting the text and the question with the answers sequentially on separate pages, without the possibility to go back and forth between the two.

Finally, OneStop includes several additional controlled experimental manipulations of reading regimes. One such manipulation teases apart ordinary reading from *information seeking*. The latter reading regime is implemented by presenting participants with the reading comprehension question prior to the paragraph. Further, each paragraph has three possible questions, with the question identity being an additional manipulation. These manipulations allow analyses of the readers’ goals with respect to the text. An additional key manipulation addresses prior exposure to the text through *repeated text presentation* within the same experimental session. OneStop includes experimental trials for each of the combinations of ordinary vs information seeking reading, Advanced vs Elementary text difficulty, and first vs second reading. The coupling of very large data, high-quality reading comprehension materials, and controlled manipulations of reading tasks, text difficulty, and repeated text presentation enables studying the multifaceted relations between eye movements, language comprehension, and different types of interaction of the reader with the text.

The unique properties of OneStop open the door for new research avenues in psycholinguistics and the psychology of reading. In particular, the exceptional scale and large number of participants in OneStop will allow new research on individual differences in reading which is currently limited by the relatively small size of existing resources. The new experimental manipulations will further drive psycholinguistics research, which, in addition to ordinary reading, will address highly common but understudied reading regimes and reader interactions with the text. OneStop will further enable new uses of eye movements data in NLP, AI, HCI and adjacent fields. These will include model interpretability and finetuning, as well as pretraining of language models on eye movements data, which was thus far hindered by the limited size of existing eye tracking datasets.

The dataset has already found novel uses in psycholinguistics, readability research, and predictive modeling of cognitive state and reading interactions using NLP and AI. These include analyses and real time prediction of information seeking^18–20^, repeated reading^21,22^, psycholinguistic benchmarking of language models^23^, evaluation of text readability measures^24^, text simplification^25^, and machine and human reading comprehension^26,27^.

## Methods

### Textual materials

The textual materials of OneStop are 30 Guardian articles, covering a diverse set of topics. The articles were obtained from the OneStopEnglish dataset^28^ (https://zenodo.org/records/1219041), which was sourced from the News Lessons section of the English language-learning portal onestopenglish.com by Macmillan Education. Permission to redistribute the articles was granted to the authors of OneStopEnglish^28^. All the texts were adjusted from British to American English. The texts are accompanied by reading comprehension questions from the OneStopQA dataset^17^ (https://github.com/berzak/onestop-qa). Each article has 4–7 paragraphs, with a total of 162 paragraphs across the 30 articles. Each article has two difficulty levels: the original Advanced Guardian text and a simplified Elementary version. The simplification was carried out manually by staff members at onestopenglish.com.

The total number of words in the OneStop articles is 19,425 in the Advanced version and 15,737 in the Elementary version. Accordingly, the mean paragraph length is 119.9 words in the Advanced version and 97.1 in the Elementary version. The mean sentence length is 20.8 words in the Advanced version and 16.9 words in the Elementary version. Each paragraph has 3 questions, corresponding to 486 questions in total. Each question can be answered based on any of the two paragraph versions. All the questions are multiple choice with 4 answer options, one of which is correct. The average question length is 9.9 words and the average answer length is 7.3 words.

OneStopQA questions have a consistent answer structure and manual annotations of auxiliary spans in the text, based on the STARC (Structured Annotations for Reading Comprehension) annotation guidelines. **A** is the correct answer. Answering a question correctly requires information from a textual span in the paragraph called the *critical span*. Typically, the critical span does not contain the answer in verbatim form. **B** is a distractor which represents a plausible miscomprehension of the critical span. **C** is a distractor which is anchored in an additional span in the paragraph, called the *distractor span*. **D** is a distractor which is plausible a priori but has no support in the text.

We note that in a small number of cases, critical and distractor spans are not continuous. The mean length of the critical spans is 31.6 words. The mean length of the distractor spans is 12.9 words.

The answers are ordered by degree of comprehension, whereby A represents correct comprehension, B reflects the ability to identify the crucial information for answering the question but failure to comprehend it correctly, C corresponds to some degree of attention to the textual content, and D provides no evidence for text comprehension with respect to the question. Two of the questions of each paragraph have identical or largely overlapping critical spans, and the third question has a distinct critical span. The correspondence between questions and critical spans is counterbalanced across the entire dataset and for each 10-article batch (see further details on the batches below) such that the number of paragraphs in which two questions share the first critical span in the paragraph is equal to the number of paragraphs in which two questions share the second critical span. Overall, this structure is well suited for studying the relations between answer choices and eye movements over their textual support. For further details on OneStopQA, see Berzak *et al*.^17^.

### Experimental design

#### Overview

In OneStop, 360 participants read the above-described Guardian articles paragraph by paragraph, and answer a reading comprehension question after each paragraph. The 30 articles are divided into three 10-article experimental batches with 54 paragraphs in each batch. Each participant reads a single batch, with a total of 120 participants reading each batch.

There are four primary manipulations in the experiment: the reading goal, the given question, the difficulty level of the text, and whether the text is presented for the first or the second time.

1. **Reading regime**: is a between subjects manipulation, where each participant is assigned to one of two reading conditions with matched textual materials: information seeking (Hunting) or ordinary reading for comprehension (Gathering). In the Hunting regime, participants are presented with the question (but not the answers) prior to reading the text. In the Gathering regime, the question is provided only after the participant has completed reading the text. The participants are distributed equally and at random across the two reading regimes, with 60 participants in each combination of a batch and a reading regime.
2. **Question**: Each participant receives one of the three possible questions for the paragraph.
3. **Text difficulty**: Each paragraph is presented either in its Advanced or Elementary version.
4. **Prior exposure to the text**: Two of the ten articles are presented for a second time, as described below.

**Fig. 1:**
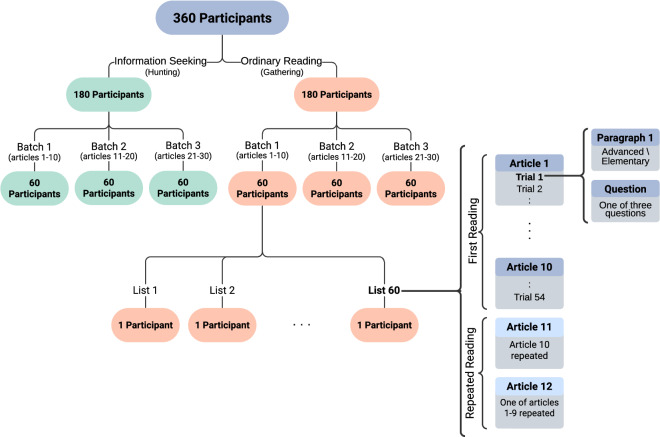
Experiment structure.

Figure 1 depicts the overall structure of the experiment.

#### Trial structure

##### Regular trials: 10 articles

A single experimental trial consists of reading a paragraph and answering one reading comprehension question about it. In the Hunting regime, a trial has 5 pages. During the presentation of page 1 (Question Preview), the participant reads the question. On page 2 (Paragraph), they read the paragraph. On page 3 (Question), they read the question again. Page 4 (Answers) retains the question and also displays the four possible answers in a cross arrangement. Participants are then required to choose one of the answers and confirm their choice. Participants are allowed to change their choice prior to confirmation. Page 5 (Feedback) informs the participants whether they answered the question correctly. Reading in the first four pages is self-paced without a time limitation. The fifth feedback page is presented for one second. The first three pages are triggered by a fixation on a target that is collocated with the first letter of the upcoming text. The target is q for page 1, P for page 2, and Q for page 3. Participants progress to the next page by pressing a button. The Gathering regime is identical to the Hunting regime, except that participants are not presented with the first Question Preview page. Consequently, participants in this condition have to be prepared for any question. Each participant is presented with a practice article consisting of two trials followed by a 10-article batch with 54 experimental trials. At the beginning of each article, a page with the article’s title is presented. Overall, the dataset includes 19,438 regular trials, 9,720 in the Hunting regime and 9,718 in the Gathering regime.

##### Repeated reading trials: 2 articles

After reading a 10-article batch, participants read two of the previously presented articles for a second time. The article in position 11 is a second presentation of the article that appeared immediately beforehand in position 10. The article in position 12 is a second presentation of an article that was presented previously in one of the positions 1–9. This corresponds to a total of 3,888 repeated reading trials, split equally between positions 11 and 12 and the two reading regimes. The second presentation of a paragraph is always with a different question than the first presentation. Each question has 8 repeated reading trials, with two appearances in each combination of reading regime and position 11 or 12.

**Fig. 2:**
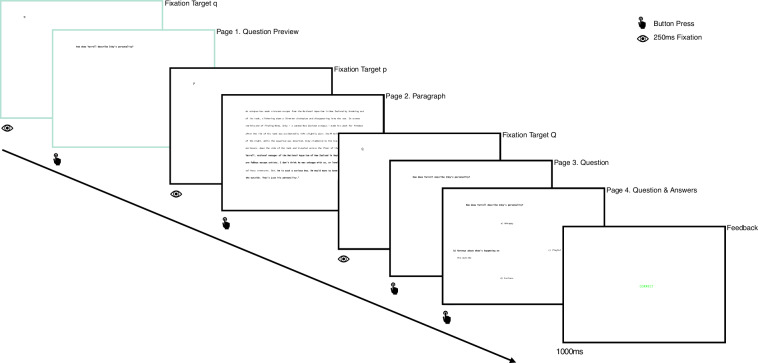
Trial structure. Pages for a single trial in the Hunting (with question preview) and Gathering (without question preview) reading regimes. Pages presented only in the Hunting regime are depicted in green.

Figure 2 depicts an example of the pages in a single trial in the Hunting and Gathering regimes.

#### Randomization and counter-balancing

In each of the 6 combinations of a batch (1 through 3) and a reading goal regime (Hunting or Gathering), each participant is assigned to one of 60 experimental lists 1–60. Each such list corresponds to one of 6 Principal lists 1–6, where *list*_*principal*_ = (*list* − 1)%6 + 1. Below, we describe the procedure for creating the Principal lists and subsequently the participant lists.

Each trial in the experiment is assigned to one of six conditions, where a condition is a combination of one of the three questions and one of the two paragraph levels. The assignment is carried out based on a Latin square design with a grid of 9 vertically tiled 6 x 6 Latin squares. Each of the 54 rows in the grid corresponds to a regular trial. The rows of each square in the grid are pre-shuffled to avoid patterns in the difficulty level of the paragraphs. The 6 columns of the grid are the 6 Principal lists of the experiment. Each batch–reading regime combination has 10 participants assigned to each Principal list. The Principal list design ensures that each participant receives an equal number of 27 paragraphs in each of the two paragraph difficulty levels. It further ensures that across the 60 participants of the batch–reading regime combination, each paragraph appears an equal number of 30 times in each difficulty version, and 20 times with each question, with 10 trials for each combination of question and paragraph difficulty level.

Principal lists serve as the basis for generating 60 participant lists, which further specify the presentation order of the articles. To generate a participant list, two articles that are to be repeated and their positions in the experiment are obtained from the pre-generated assignment of repeated articles described below. Then, the presentation order of the articles in the remaining eight positions is randomized. The answer order is also randomized for each question instance. The randomization of the article order and answer order is identical across the two reading conditions for each batch, and thus the lists in each of the two reading regimes of each batch are identical.

Repeated articles appear with identical paragraph levels as in the first reading, but with different questions according to a question transition matrix, the application of which retains Latin square properties for the repeated reading articles. Articles are selected to be repeated at random, under the following counter-balancing constraints in each combination of a batch and a reading goal regime. The first constraint is that each article appears an equal number of 6 times in each of the positions 11 and 12. A second constraint is that articles repeated in position 12 are distributed such that 6 articles appear in each of the positions 2 through 9 and 12 articles appear in position 1. Additionally, an article that is repeated in position 12 cannot appear more than once in any single position from 2 to 9. Across the entire dataset, this corresponds to 360 article appearances in position 10 that are presented for a second time in position 11, and 360 article appearances in positions 1 to 9 that are presented for a second time in position 12, with 72 articles in position 1 and 36 articles each in positions 2 through 9. We pre-generated a single 60-participant assignment of repeated article IDs and their positions in the first and second article presentations, which is used in all 6 batch–reading goal regime combinations.

### Participants

The participants in OneStop are 360 L1 speakers of English. We considered a participant as an L1 speaker of English if they met the following three criteria: (1) the participant self-identified as a native speaker of English, (2) English Age of Acquisition (AoA) of 4 or younger, and (3) the participant spent most of their life in an English speaking country. We generally tried to avoid recruiting participants with dyslexia, language impairments, and eye conditions. 303 participants were recruited in the Boston area US and 57 in Haifa Israel. Participants were recruited through human subject mailing lists, social media advertisements, flyers on physical message boards, and others. Participants received a monetary compensation of $25 in Boston and 100 NIS in Haifa. We excluded participants who did not complete the experiment, most commonly due to tracking or calibration issues.

#### Ethics approval and consent to participate

The study was approved under IRB protocols 1605559077 (MIT) and 157-2022 (Technion). All the participants provided written consent for participation and de-identified data sharing on a physical consent form prior to commencing the experiment.

### Procedure

Participants were invited to the lab based on their responses to an eligibility questionnaire. Participants with corrected vision were asked to wear contacts for the experiment. The experiment took place in an acoustically shielded room, with the experimenter present in the room. Participants were first consented, then filled out the participant questionnaire described below, and did an eye dominance test. At the beginning of the eye tracking experiment, participants were instructed to read silently and to minimize head movement. The experiment started with two instruction pages, following which the experimenter walked the participant through the first trial. The experiment included two breaks, one at the end of the 4th article and one at the end of the 8th article. Before the 11th article, a dedicated page informed the participants that they were about to read two articles that they had read previously. Throughout the session, experimenters followed an experiment script to ensure that instructions regarding all aspects of the experiment are conveyed similarly across participants and experimenters.

### Eye tracking setup

The physical setup described below was identical in both data collection sites.

#### Eye tracker

We used a Tower Mount Eyelink 1000 Plus eye tracker (SR Research) at a sampling rate of 1000 Hz. Eye movements were recorded for the participants’ dominant eye. In a small number of cases, experimenters switched to the non-dominant eye during the course of the experiment due to tracking or calibration issues.

#### Monitor

The experiment was presented on a 27 inch monitor (Dell U2715H) with a display area of 597 mm × 336 mm, resolution of 2560 px × 1440 px and refresh rate of 60 Hz. Participants’ eye level was 750 mm away from the top of the monitor’s display area and 795mm away from its bottom. In this setup, participants’ eyes were about 45 mm below the top of the monitor’s display, approximately at the same height as the top most position of the text.

#### Controller

Participants used a Logitech Gamepad F310 controller during the experiment. The button A was used for proceeding to the next page after finishing reading, as well as for confirming the answer selection. The four buttons of the directional pad were used for choosing answers.

### Text presentation

We used the Lucida Sans Typewriter monospace font, with a font size of 25 pt (each letter occupying 19 px × 38 px). Horizontally, this corresponds to approximately 0.34 per letter. We used triple spacing (76 px) between lines. The relatively large spacing aims to reduce assignment of fixations to incorrect lines. The top left position of the questions and the paragraphs was (300, 186) with a text area width of 1824 px (96 characters). Questions were 1–2 lines and paragraphs were 3–10 lines. Answers were presented in a cross arrangement which naturally maps to the four buttons of the directional pad. The answer text area width was 700 px, and the answers were 1–3 lines.

### Calibration

OneStop implemented a stringent protocol with a low calibration validation approval threshold, combined with an automated mechanism for detecting drift and triggering calibration in response, as described below.

#### Procedure

We used 9 point calibration with bulls-eye targets (18 px outer circle, 6 px inner circle). During the first calibration, participants were requested to look at the center of the targets, which in our experience had a positive impact on the calibration precision. Calibration was performed at least 3 times during the experiment: once at the beginning of the experiment and once after each of two breaks. Calibration quality was monitored throughout the experiment, and calibration was also performed upon failure to trigger the text at the beginning of a trial which indicated gaze drift as described below. The experimenters were instructed to repeat calibration until an average validation error below 0.3 was reached.

#### Text triggering

Prior to the presentation of the question preview, paragraph and question, participants were presented with a page presenting a fixation target located at (300, 186), the same position as the first letter of text on the following page. The targets were **q** for the question preview, **p** for the paragraph and **Q** for the question. The presentation of the following text page was triggered by a fixation of at least 250 ms within a 39 px × 48 px rectangular area centered around the 19 px × 38 px area of the target letter. This corresponds to a horizontal margin of about half a letter width, and a vertical margin of about a quarter of a line space around the target letter. To prevent the possibility of skipping a page due to an accidental button press, button presses were locked for 1 second after the presentation of a question, and for 2 seconds after the presentation of a paragraph.

#### Drift monitoring and recalibrations

We implemented the following calibrate-as-needed automatic procedure for initiating calibrations during the experiment. Failure to produce a 250 ms fixation within 4 seconds on the first target of the trial (**q** target in the Hunting condition and **p** target in the Gathering condition), automatically triggered a calibration session. Note that this procedure automates drift monitoring and calibration triggering, relieving experimenters from these duties and reducing the potential for collecting data with drift due to experimenter oversight. In order to avoid calibration in the middle of a trial, for subsequent trial targets (**p** and **Q** in the Hunting condition and **Q** in the Gathering condition), the next page was presented even if the participant was not able to produce a 250 ms fixation on the target letter within 4 seconds.

### Experiment software

The experiment was implemented and deployed using the SR Research Experiment Builder software.

### Data processing

The eye tracking data is released in four different formats, corresponding to different types of preprocessing and information aggregation. We provide the raw data of the entire experiment duration of each participant in .edf format. We further provide this data as ASCII files generated using the edf2asc utility script (v4.2.1.0) by SR Research. We also release Interest Area and Fixation reports produced using the SR Data Viewer (4.3.210), which group the raw data into fixations and saccades, and include all the features available in Data Viewer for these report types. Separate reports are generated for seven Interest Periods in the experiment: Article Title, Question Preview (page 1), Paragraph (page 2), Question (page 3), Answers (page 4), QA (pages 3 + 4), and Feedback (page 5). Interest Areas in the Data Viewer reports span whitespace tokenized words.

In addition to the eye movement features available in SR Data Viewer, we provide additional variables that encode experiment and trial information. We further include annotations for the “big three” word properties^2,29,30^: frequency, surprisal and word length, as well as syntactic, morphological and Named Entity information extracted using spaCy^31^. In the Paragraph reports, we also include annotations of word membership in the critical and distractor spans according to the annotations of OneStopQA. All the annotations are generated using the process_sr_report.py script and described further below. We further release summary statistics and information on each participant’s experiment session. This summary is generated using the compute_session_summary.py script.

## Data Records

### Data repository

The eye-tracking data and anonymized participant information are available in an Open Science Framework (OSF) repository^16^ at https://osf.io/2prdq/. The OSF data repository includes the following components that support different use cases of the data.

- **OneStop Ordinary Reading**^32^ (https://osf.io/zn9sq/): Paragraph Interest Period for the ordinary reading part of the data.
- **OneStop Information Seeking**^33^ (https://osf.io/kpbgx/): Paragraph Interest Period for the information seeking part of the data.
- **OneStop Repeated Reading**^34^ (https://osf.io/4ay3t/): Paragraph Interest Period for the repeated reading part of the data.
- **OneStop Information Seeking in Repeated Reading**^35^ (https://osf.io/6ra7t/): Paragraph Interest Period for the repeated reading in information seeking part of the data.
- **OneStop All Regimes**^36^ (https://osf.io/azj2g/): Paragraph Interest Period for the entire dataset.
- **OneStop Full**^37^ (https://osf.io/z7pyn/): Complete trials for the entire dataset, divided into the following seven Interest Periods: Title, Question Preview, Paragraph, Question, Answers, QA (Question + Answers) and Feedback.
- **OneStop Raw Data**^38^ (https://osf.io/6f2km/): Complete trials for the entire dataset, in EDF and ASCII formats.
- **OneStop Metadata**^39^ (https://osf.io/jbd24/): Participant questionnaire and experiment summary statistics.

### Data files

We release the following data files, listed below by dataset component.

Components 1–6: OneStop Ordinary Reading, OneStop Information Seeking, OneStop Repeated Reading, OneStop Information Seeking in Repeated Reading, OneStop, OneStop Full.

- Fixation Reports: Eye movement features, experiment and trial information, and linguistic word properties aggregated at the level of individual fixations, in tab-separated CSV format.
- Interest Area Reports: Eye movement features, experiment and trial information, and linguistic word properties aggregated at the word level, in tab-separated CSV format.

Table 1 lists the data files of the Fixation Reports and Interest Area reports by Interest Period. Note that while all the listed files are included in OneStop Full, the other five dataset components contain reports only for the Paragraph Interest Period.

**Table 1:** Fixation and Interest Area report files.

Interest Period	Page	Content	Fixation Report	Interest Area Report
Title	Title	article title	fixations_Title.csv	ia_Title.csv
Question Preview	1	question	fixations_Question_preview.csv	ia_Question_preview.csv
Paragraph	2	paragraph	fixations_Paragraph.csv	ia_Paragraph.csv
Question	3	question	fixations_Question.csv	ia_Question.csv
Answers	4	question and answers	fixations_Answers.csv	ia_Answers.csv
QA	3+4	question and answers	fixations_QA.csv	ia_QA.csv
Feedback	5	CORRECT / INCORRECT	fixations_Feedback.csv	ia_Feedback.csv

Component 7: OneStop Raw Data.

- l[list]_[subj_id].edf: The raw eye tracking data for the entire eye tracking session of each participant, in EDF format.
- l[list]_[subj_id].asc: Gaze location and additional features at 1ms intervals for the entire eye tracking session of each participant, in ASCII format.

Component 8: OneStop Metadata.

- questionnaire.json: Anonymized participant demographics and language history questionnaire.
- session_summary.csv: Summary statistics and information on each participant’s experiment session, such as reading comprehension accuracy and experiment duration.

### Data variables

The eye movement features in the Fixation Reports and Interest Area Reports are documented in the Data Viewer user manual. Additional variables for the experiment, trials and linguistic annotations are listed in Table 2. The data variables of the participant questionnaire file are listed in Table 3. Table 4 provides the variables included in the session summary file.

**Table 2:** Experiment and trial variables, and linguistic annotations in the Fixation and Interest Area reports.

Category	Feature	Description	Values
Experiment Variables	participant_id	Participant’s ID	String (360 unique values)
	list_number	Experimental list	1 - 60
	question_preview	Was the question presented before the paragraph (i.e. the reading regime)	True / False
	article_batch	A 10-article batch assigned to the participant	1 (articles 1–10) / 2 (articles 11-20) / 3 (articles 21-30)
Trial Variables	trial_index	The per-participant trial index	1 –70 (last trial)
	practice_trial	Whether the trial was a practice trial	True / False
	article_id	The unique identifier for an article in a batch. Article 0 is the practice article	0-10
	paragraph_id	The unique identifier for a paragraph in an article	1–7
	difficulty_level	Paragraph difficulty level	Adv / Ele
	repeated_reading_trial	Whether the trial was a repeated reading trial	True / False
	article_index	The index of the article in the session. Article 0 is the practice article	0–12
	article_title	The article title, presented before the first paragraph of each article	String
	paragraph	The paragraph presented in the trial	String
	question	The question presented in the trial	String
	onestopqa_question_id	The unique identifier for the question presented in the trial	0–2
	same_critical_span	Whether there was another question with the same critical span	0 if no other question. 1 or 2 otherwise (arbitrarily).
	selected_answer	The answer selected by the participant	A/B/C/D
	is_correct	Whether the participant selected the correct answer (A)	True / False
	selected_answer_position	The position on the page of the answer selected by the participant	0/1/2/3 corresponding to: top, left, right, bottom
	correct_answer_position	The position on the page of the answer correct answer for the trial	0/1/2/3 corresponding to: top, left, right, bottom
	answers_order	Mapping between position on page and A/B/C/D	list of ABCD corresponding to: top, left, right, bottom
	answer_1	The answer presented in the trial in the top position	String
	answer_2	The answer presented in the trial in the left position	String
	answer_3	The answer presented in the trial in the right position	String
	answer_4	The answer presented in the trial in the bottom position	String
Linguistic Annotations Big Three	word_length	Number of characters in the word	Integer
	word_length_no_punctuation	Number of characters in the word excluding punctuation	Integer
	subtlex_frequency	$-{\log }_{2}$ word frequency from SUBTLEX-US^47^	Bits
	wordfreq_frequency	$-{\log }_{2}$ word frequency from Wordfreq^44^	Bits
	gpt2_surprisal	Surprisal from GPT-2^43^	Bits
Linguistic Annotations Universal Dependencies (UD)^48^	universal_pos	Universal part-of-speech tag	17 possible tags
	ptb_pos	Penn Treebank part-of-speech tag	See label scheme here
	head_word_index	Index of the syntactic head word in the dependency tree	Integer, 0 for root - number of words in the sentence
	dependency_relation	Dependency relation label to the head word in the dependency tree	See label scheme here
	left_dependents_count	Number of syntactic dependents to the left	Integer
	right_dependents_count	Number of syntactic dependents to the right	Integer
	distance_to_head	Distance in words to the syntactic head	Integer, starting at 1 for adjacent words
	morphological_features	List of morphological features of the word	See list here
	entity_type	The entity type of the word (if applicable)	See label scheme here. *None* if not an entity.
STARC Auxiliary Spans^17^	auxiliary_span_type	Whether a word is part of the critical span or the distractor span	critical / distractor / outside
	critical_span_indices	Start and end word indices of the critical span	list of tuples of integers
	distractor_span_indices	Start and end word indices of the distractor span	list of tuples of integers
ANSWER_LOCATIONS	PREVIEW_QUESTION_RT	The total reading time of the preview question	Integer
	PARAGRAPH_RT	The total reading time of the paragraph	Integer
	QUESTION_RT	The total reading time of the question	Integer
	ANSWER_RT	The total reading time of the answers	Integer
	SELECT_FINAL_ANSWER_RT	The time from the answers display to the final answer press	Integer
	SELECT_TO_CONFIRM_RT	The time from choosing the final answer to confirming it	Integer
	CONFIRM_FINAL_ANSWER_RT	The time from the answers display to answer confirmation.	Integer
	ANSWER_PRESS_NUMBER	The total number of answer selection button presses	Integer
	EXPERIMENT_DURATION	Accumulative time from the beginning of the experiment	Minutes (Integer)
	NUM_RECALIBRATIONS	An accumulative number of recalibrations during the experiment	Integer

UD annotations are extracted using spaCy^31^. See the SR Data Viewer user manual for documentation of eye movement variables in these reports. Note, missing values are denoted by “.”.

**Table 3:** Participant questionnaire variables.

Variable	Description	Values
Participant ID	Participant’s ID	String (360 unique values)
Age	Participant’s age	In years
Gender	Participant’s gender	Male / Female / Other
Home Country	Participant’s home country	List of countries
Education Level	Highest or current level of education	Secondary or High school / College or University Undergraduate / College or University Postgraduate
Years in Secondary/High School	Number of years spent in secondary/high school	Numeric
Years in Undergraduate	Number of years spent in college/university undergraduate	Numeric
Years in Postgraduate	Number of years spent in college/university postgraduate	Numeric
University Affiliation	Whether affiliated with or a student at a university	Yes / No
University Institution	Institution the participant is affiliated with, if applicable	MIT / Tufts / Harvard / BU / Other
University Role	Role at the institution, if affiliated with a university	Undergraduate student / Graduate student / Postdoc / Research staff / Faculty / Administrator / Other
Native English Speaker	Whether the participant considers themselves to be a native English speaker	Yes / No
English AoA	English Age of acquisition	Since birth, or numeric age in years
Countries Lived In	Countries lived in for more than a month, with from/to dates	List of countries with from/to month and year for each
	Reading habits* in English	
Textbooks	Time spent per week reading textbooks	0 to 7+ hours
Academic	Time spent per week reading academic materials other than textbooks	0 to 7+ hours
Magazines	Time spent per week reading magazines	0 to 7+ hours
Newspapers	Time spent per week reading newspapers	0 to 7+ hours
Email	Time spent per week reading email	0 to 7+ hours
Fiction	Time spent per week reading fiction books	0 to 7+ hours
Nonfiction	Time spent per week reading nonfiction/special interest books	0 to 7+ hours
Internet	Time spent per week reading internet media (all categories not included above)	0 to 7+ hours
Other	Time spent per week reading other categories	0 to 7+ hours
	For each additional language spoken other than English	
Language	Name of the language	Language names from WALS^45^
Language Proficiency	Overall proficiency level	Beginner / Intermediate / Advanced / Near-native / Native
Speaking Proficiency	Speaking proficiency	1-10 scale
Understanding Proficiency	Understanding spoken language proficiency	1-10 scale
Reading Proficiency	Reading proficiency	1-10 scale
Language AoA	Age at which started learning the language	Since birth, or numeric age in years
Language Learning Duration	Number of years learning and /or using the language	Numeric
	Reading habits* in additional language if Language Proficiency is Native	
Textbooks	Time spent per week reading textbooks	0 to 7+ hours
Academic	Time spent per week reading academic materials other than textbooks	0 to 7+ hours
Magazines	Time spent per week reading magazines	0 to 7+ hours
Newspapers	Time spent per week reading newspapers	0 to 7+ hours
Email	Time spent per week reading email	0 to 7+ hours
Fiction	Time spent per week reading fiction books	0 to 7+ hours
Nonfiction	Time spent per week reading nonfiction/special interest books	0 to 7+ hours
Internet	Time spent per week reading internet media (all categories not included above)	0 to 7+ hours
Other	Time spent per week reading other categories	0 to 7+ hours
	Conditions	
Dyslexia	Presence of dyslexia	No / Dyslexia
Language Impairments	Presence of language impairments	No / Language impairment (free text)
Eye Conditions	Presence and type of eye conditions	No / Yes (Amblyopia, Astigmatism, Keratoconus, Nystagmus, Strabismus, Other)

* Reading habits questions are based on Section 1 of the reading habits self-report of Acheson *et al*.^46^.

**Table 4:** Session summary file variables.

Variable	Description	Values
participant_id	Participant’s ID	String (360 unique values)
article_batch	A 10-article batch assigned to the participant	1 (articles 1–10) / 2 (articles 11–20) / 3 (articles 21–30)
list_number	Experimental list	1–60
question_preview	Was the question presented before the paragraph (i.e. the reading regime)	True / False
data_collection_site	Location of data collection	MIT / Technion
comprehension_score-regular_trials	Participant’s overall reading comprehension score during first reading (10 articles, 54 regular trials)	0–100%
comprehension_score-repeated_reading	Participant’s overall reading comprehension score during repeated reading (2 articles, 8-14 repeated trials)	0–100%
recalibration_count	Number of times the session was interrupted to recalibrate the eye tracker	0 or more
total_recalibrations	Number of times the eye tracker was recalibrated during the session (in addition to the 3 obligatory calibrations)	0 or more
mean_validation_error	Mean validation error across all calibrations immediately preceding text reading	visual degrees
total_session_duration	Total duration of the experimental session (including breaks and calibrations)	minutes
session_duration	Duration of the experimental session excluding breaks and calibrations	minutes
dominant_eye	Participant’s dominant eye	L / R
tracked_eye	Eye that was tracked (typically the dominant eye)	L / R / LR **
lextale_score	Participant’s score on the LexTALE vocabulary test *	0–100

* LexTale scores are available for 100 participants. ** L: left eye, R: right eye, LR: data was collected from both eyes (switched between eyes during the experiment).

## Technical Validation

We present results for four analyses that characterize the collected eye tracking data and validate its quality. The first analysis includes data statistics. The second analysis examines data quality. In the third analysis, we present benchmarks for standard eye movement measures. Our final analysis replicates a key finding in the psycholinguistic literature, namely a sensitivity of reading times to the linguistic properties of the text. The analyses include comparisons to publicly available English eye tracking for reading datasets with L1 speakers.

### Key statistics

#### Collected data, compared to existing English L1 datasets

Overall, the dataset includes eye movement recordings for 3,982,709 word tokens: 38,798 in the article titles, 121,110 during question preview (page 1), 2,632,159 while reading the article paragraphs (page 2), and 1,190,732 during question answering (pages 3 and 4). See the Methods Section for descriptions of the experiment pages. Table 5 presents a comparison of key statistics in OneStop against the currently publicly available broad coverage English eye tracking datasets with L1 participants. The datasets are divided into three categories: (i) datasets for standardized or extensively piloted reading comprehension materials, (ii) general purpose datasets with full passages (iii) general purpose datasets with single sentences. The comparison indicates that OneStop has more participants and more word tokens recorded than all the currently existing datasets combined. OneStop is also the largest dataset with respect to the number of reading comprehension questions, with over 20 times more questions than SB-SAT. The OneStop corpus size is larger than most of the other datasets, but is smaller than Dundee, GECO, and CELER.

**Table 5:** Statistics of publicly available English L1 datasets for passage reading.

	Dataset	Subjs	Age	Words	Words Recorded	Qs	Subjs per Q	Qs per Subj
**RC**	OneStop	360	22. 8_±5.6_	19,428 (Adv); 15,737 (Ele)	2,632,159 (Parag)	486	20	54
				19,221 (QA)	1,311,752 (QA)			
	SB-SAT L1^6^	66	NA	2,539	167,574	20	95	20
**Passage**	Dundee^1^	10	NA	51,502	307,214	NA	10	NA
	GECO L1^3^	14	21. 8_±5.6_	56,410	774,015	NA	14	NA
	Provo^4^	84	NA	2,689	225,624	0	0	0
	MECO En^8^	46	21. 0_±2.2_	2,109	83,246	48	46	48
**Sent**.	CELER L1^7^	69	26. 3_±6.7_	61,233	122,423	78^*^	69	78
	ZuCo^49^	18	34. 3_±8.0_	15,138	272,484	42^**^	18	42
	UCL^50^	43	25. 8_±7.5_	1,932	81,144	110	43	110

"RC” (Reading Comprehension) are datasets for piloted reading comprehension materials. The remaining datasets are general purpose broad coverage datasets for passages and individual sentences. “Words” is the number of words in the textual corpus. Note that unlike the other datasets where each participant reads the entire corpus, in OneStop each participant reads about a third of the corpus, 4225.15 (*SD* = 148.9) words on average. “Words Recorded” is the number of word tokens for which eye tracking data was collected. NA: data not available. Standard deviation in parentheses. ^*^ In 5,283 additional tasks in CELER (78 per participant), participants are required to answer whether a given word appeared in the sentence. ^**^ In 390 additional tasks in ZuCo, participants are asked to determine whether the sentence contains a specific semantic relation.

#### Participant characteristics

**Fig. 3:**
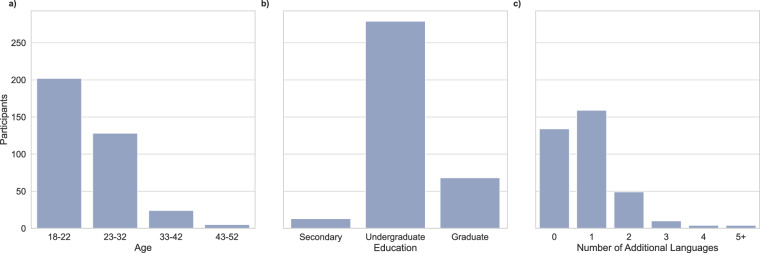
Participant characteristics. (**a**) Age (**b**) Level of education (**c**) Number of additional languages spoken.

Out of the 360 participants, 238 identified as female, 117 as male, and 5 as other. The mean participant age is 22.8. 29 of the participants are balanced bilinguals. The mean English Age of Acquisition (AoA) is 0.4. Seven participants in the dataset have an AoA older than four, and eleven spent less than half of their life in an English speaking country. 289 (80%) of the participants have a university affiliation, and 264 are students (73%). The dataset includes 1 participant with mild dyslexia and 1 participant with a mild reading and writing impairment diagnosed during childhood. 22 participants have astigmatism, 18 lens-corrected myopia, 3 astigmatism and myopia, 1 amblyopia, and 2 have an unspecified eye condition. Figure 3 presents participant distributions for age, level of education and number of additional languages spoken.

#### Experiment duration

**Fig. 4:**
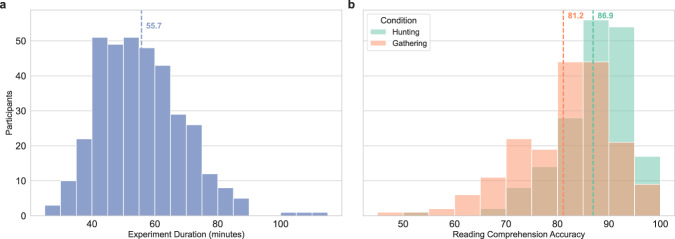
Experiment duration and reading comprehension performance. (**a**) Experiment duration including breaks and calibration sessions (**b**) Participant reading comprehension accuracy (% correct out of all 54 reading comprehension questions in the regular trials) by reading regime: information seeking (Hunting) and ordinary reading for comprehension (Gathering). Vertical dotted lines represent mean values.

The mean experiment duration, including breaks and calibrations, was 55.7 minutes, 54.3 minutes in the information seeking (Hunting) regime and 57.1 minutes in the ordinary reading (Gathering) regime. Figure 4(a) presents the distribution of experiment duration across participants. The mean total reading time (i.e. experiment duration excluding breaks and calibrations, and including practice items) across participants was 44.3 minutes, 43.3 minutes in the Hunting regime and 45.3 minutes in the Gathering regime. The mean total reading time for articles (excluding titles) was 26 minutes, 23.1 minutes in the Hunting regime and 28.9 minutes in the Gathering regime. The mean total time reading and answering questions was 10.2, 13.1 minutes in the Hunting regime (of which 3.5 minutes were for question preview) and 10.8 minutes in the Gathering regime.

#### Reading comprehension performance

The reading comprehension accuracy in the regular trials is 86.9% in the Hunting regime, and 81.2% in the Gathering regime, which is lower (*p* < 10^−9^). The reading comprehension accuracy in the repeated reading trials is 90.6% in the Hunting regime and 84.2% in the Gathering regime, which is higher than in the first reading trials in both regimes (*p* < 10^−3^). Fig. 4(b) presents a histogram of participant reading comprehension scores. We note that there is no statistically significant relationship between reading comprehension and reading speed (mean per-word Total Fixation duration) in regular trials (*R* = 0.001, *p* = 0.53).

### Data quality

#### Calibration quality

**Fig. 5:**
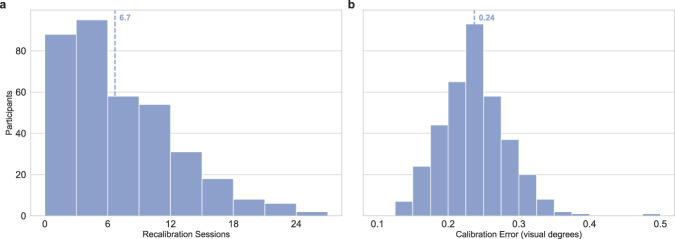
Per participant calibration statistics. (**a**) Number of re-calibration sessions, in addition to the three obligatory calibration sessions (**b**) Mean calibration validation error for calibrations immediately preceding text reading. Vertical dotted lines represent mean values.

The mean number of calibration sessions during an experiment due to failures to trigger fixation targets at the beginning of trials is 6.7. The mean number of calibration attempts per calibration session was 1.3. The mean calibration validation error across all the calibrations in the dataset immediately preceding text reading is 0.24 (*SD* = 0.05), which corresponds to about 7/10 width of a single letter. 15.5% of these calibrations had a mean validation error above the 0.3 threshold provided to the experimenters as the maximum target validation error. Fig. 5 presents the distributions of the number of recalibration sessions and mean validation error per participant. Overall, the dataset strikes a good balance between very high calibration accuracy and a reasonable number of calibration sessions during the experiment.

#### Accuracy of fixation assignments to lines

We conducted an additional quality assurance analysis which quantifies the prevalence of erroneous line assignments of fixations due to vertical drift. To this end, we defined an annotation scheme for classifying fixations into three categories: line-reading fixations assigned to the correct line, line-reading fixations assigned to an incorrect line, and other. The other category includes return sweep completion sequences, fixations outside of an interest area, and fixations that are shorter than 50 ms or longer than 500 ms. To carry out annotations according to these categories, we created trial scanpath sequence visualizations where fixations are numbered and color coded according to their line assignment. Fixations shorter than 50 ms or longer than 500 ms, and fixations outside interest areas were automatically marked with a designated color.

Two of the paper’s authors independently annotated fixation sequence visualizations of 20 regular trials, each with 10 lines (1,296 fixations in total), according to the above scheme. Each trial was taken from a different randomly chosen participant in the ordinary reading regime, such that it is the trial with the smallest number of fixations among the 10-line trials read by the participant. This trial selection procedure is conservative in that it covers the maximum screen area used in experiment trials. At the same time, we use only regular, ordinary reading trials with the smallest number of fixations. Such trials tend to have less clutter, rereading, line skipping and irregular reading behavior, facilitating the annotation of fixation categories.

On average across the two annotators, 76.2% of the fixations were marked as line reading and 23.8% as other (21.4% return sweep completions, no fixations outside interest areas, and 2.4% fixations that are shorter than 50ms or longer than 500ms). Crucially, only 3.7% of the total number of fixations were marked by both annotators as line fixations with an incorrect line assignment, and 3.9% were marked as such by at least one annotator. The agreement rate between the two annotators in assigning fixations to the three fixation categories was high, with a Cohen’s Kappa of 0.95. This outcome suggests that the combination of triple spacing between lines, a high quality eye tracker, drift-conditioned triggering of calibrations and a low target validation error, leads to a small number of line assignment errors, further validating the high quality of the collected eye movement data. The annotation guidelines, fixation sequence visualizations, and fixation annotations are available in the GitHub repository of the dataset.

### Benchmarks for global eye movement measures

Next, we examine several key standard reading time measures from the psycholinguistic literature.

- Average Fixation Duration: the average duration of a fixation.
- First Fixation Duration: the duration of the first fixation on a word.
- Gaze Duration: the time from first entering a word to first leaving it.
- First Pass Gaze Duration: the time from first entering a word to first leaving it during first pass reading.
- Total Fixation Duration: the sum of all fixation durations on the word.
- Regression Rate: the fraction of backward saccades out of the total number of saccades originating from words that were fixated.
- Fixation Count: average number of fixations per word.
- Skip Rate: the fraction of words that were skipped.
- First Pass Skip Rate: the fraction of words that were skipped during first pass reading.
- Saccade Length: the average length of a saccade.

Table 6 presents statistics of standard eye movement measures for OneStop in the regular trials of the Gathering regime. We further provide these measures in SB-SAT, and the two largest datasets with respect to corpus size in the sentence and passage reading categories, CELER L1 and GECO L1. In CELER L1, comprehension is tested after each sentence. In GECO L1, it is tested after each chapter of a book.

**Table 6:** Means with 95% confidence intervals for eye movement measures in OneStop (for paragraphs in ordinary reading, regular trials), SB-SAT, CELER L1 and GECO L1.

Measure	OneStop	SB-SAT	CELER L1	GECO L1
Average Fixation Duration	191. 6_±3.7_	194. 9_±6.1_	213. 1_±6.0_	NA
First Fixation Duration	193. 7_±3.9_	215. 1_±6.3_	212. 3_±6.2_	211. 8_±14.4_
Gaze Duration	214. 6_±5.2_	260. 9_±11.3_	246. 0_±8.6_	233. 9_±17.6_
First Pass Gaze Duration	218. 4_±5.4_	219. 0_±6.6_	250. 3_±8.8_	237. 9_±18.6_
Total Fixation Duration	306. 6_±10.9_	360. 0_±18.8_	361. 7_±21.2_	274. 2_±24.9_
Regression Rate	0.23_±0.01_	NA	0.27_±0.03_	NA
Fixation Count	1.04_±0.05_	NA	1. 3_±0.1_	0. 8_±0.1_
Skip Rate	0.36_±0.01_	NA	0.26_±0.02_	0.39_±0.03_
First Pass Skip Rate	0.55_±0.02_	NA	0.36_±0.02_	0.52_±0.04_
Saccade Length (Visual Angle)	5. 0_±0.1_	4. 4_±0.2_	3. 1_±0.2_	NA
Saccade Length (# Characters)	14. 8_±0.4_	4. 4_±0.2_	8. 7_±0.4_	NA

First Fixation, Gaze Duration, First Pass Gaze Duration and Total Fixation Duration times exclude words that were not fixated. The mean value of each measure is the intercept of a linear mixed-effects model *Measure* ~ 1 + (1|*subj*) + (1|*item*) where item is a paragraph in OneStop, a sentence in CELER, and a novel in GECO. We mark “NA” for measures which cannot be computed from the public releases of the dataset.

We find that the measures are largely comparable across datasets. As can be expected, OneStop measures tend to be closer to the passage reading data of GECO L1 than to the sentence reading data of CELER L1. SB-SAT has a longer Total Fixation Duration compared to OneStop (*p* < 10^−7^) and GECO L1 (*p* < 10^−7^), which could be related to the possibility to return to the text after having read the questions in this dataset.

### The effect of linguistic word properties on reading times

Effects of word predictability, frequency, and length on reading times are ubiquitously present in eye movements corpora^40–42^ among others. Our final analysis replicates these effects in OneStop. We quantify predictability using surprisal, defined as $-\log p({w}_{i}| {w}_{ < i})$, where *w*_*i*_ is the current word and *w*_<*i*_ is the preceding context. We use surprisal estimates from the GPT-2 language model^43^, and log frequency $-\log p({w}_{i})$ estimates from Wordfreq^44^. Both estimates are released with the data (see Table 2).

Table 7 presents these effects for First Fixation duration, Gaze Duration, and Total Fixation duration. We further provide these effects in CELER L1 and GECO L1^7^. We observe that similarly to these datasets, surprisal, frequency and length effects are present across all three measures in OneStop.

**Table 7:** The effects of current word surprisal, frequency and word length on reading times, with 95% confidence intervals.

	OneStop			CELER L1			GECO L1		
	FF	GD	TF	FF	GD	TF	FF	GD	TF
Intercept	$195.{2}_{\pm 2.1}^{* * * }$	$212.{9}_{\pm 2.7}^{* * * }$	$302.{0}_{\pm 5.6}^{* * * }$	$214.{0}_{\pm 6.3}^{* * * }$	$236.{9}_{\pm 7.7}^{* * * }$	$345.{8}_{\pm 20.4}^{* * * }$	$210.{7}_{\pm 22.3}^{* * * }$	$227.{2}_{\pm 26.2}^{* * * }$	$264.{6}_{\pm 40.8}^{* * * }$
Frequency	$1.{0}_{\pm 0.1}^{* * * }$	$1.\,{1}_{\pm 0.1}^{* * * }$	$2.{0}_{\pm 0.3}^{* * * }$	$1.{3}_{\pm 0.4}^{* * * }$	$1.{9}_{\pm 0.5}^{* * * }$	$3.{2}_{\pm 1.0}^{* * * }$	$1.{1}_{\pm 0.3}^{* * * }$	$1.{4}_{\pm 0.5}^{* * * }$	$1.{4}_{\pm 1.0}^{* * }$
Surprisal	$1.{2}_{\pm 0.1}^{* * * }$	$2.{3}_{\pm 0.1}^{* * * }$	$6.{7}_{\pm 0.4}^{* * * }$	$0.{6}_{\pm 0.3}^{* * * }$	$1.{9}_{\pm 0.7}^{* * * }$	$6.{7}_{\pm 1.0}^{* * * }$	$0.{6}_{\pm 0.1}^{* * * }$	$1.{1}_{\pm 0.2}^{* * * }$	$3.{0}_{\pm 0.8}^{* * * }$
Length	$-0.{5}_{\pm 0.1}^{* * * }$	$3.{5}_{\pm 0.3}^{* * * }$	$12.{7}_{\pm 0.7}^{* * * }$	$-0.{8}_{\pm 0.9}^{* * * }$	$4.{3}_{\pm 1.5}^{* * * }$	$14.{5}_{\pm 2.5}^{* * * }$	$-0.{2}_{\pm 0.5}^{(.)}$	$4.{5}_{\pm 1.9}^{* * * }$	$8.{6}_{\pm 2.4}^{* * * }$

FF = First Fixation; GD = Gaze Duration; TF = Total Fixation Duration. Depicted are current word coefficients from a linear mixed-effects model for each measure and dataset, that predict reading times from these properties of the current and previous words *RT* ~ *freq* + *len* + *surp* + *freq*_*prev*_ + *len*_*prev*_ + *surp*_*prev*_ + (*freq* + *len* + *surp*|*subj*) + (*freq* + *len* + *surp*|*item*). *prev* refers to the previous word, to account for spillover effects^51^.

All the predictors are centered. Previous word properties were omitted from the random effects structure due to model convergence issues. Following common practice, we exclude out-of-vocabulary words, skipped words, words with punctuation, numbers and words that begin or end a page. Values for CELER L1 and GECO L1 are taken from Berzak *et al*.^7^. ***p < 0.001, **p < 0.01, *p < 0.05, (.)’ *p* ≥ 0.05. Tests performed using the MixedModels library in Julia^52^.

## Usage Notes

The underlying textual materials and auxiliary text annotations of OneStopQA are provided under the OneStopQA Creative Commons Attribution-ShareAlike 4.0 International License. The eye tracking data and anonymized participant questionnaire responses are released under a Creative Commons Attribution 4.0 International License.

## Data Availability

The OneStop dataset^16^, including all the dataset components^32–39^, is available in an Open Science Framework (OSF) repository at https://osf.io/2prdq/. Detailed documentation of the data, the fixation line assignment annotations, as well as preprocessing and analysis scripts are available in a GitHub repository at https://github.com/lacclab/OneStop-Eye-Movements.

## Code availability

The code for this paper is publicly available at https://github.com/lacclab/OneStop-Eye-Movements.

## References

[1] Kennedy, A., Hill, R. & Pynte, J. The Dundee corpus. In *Proceedings of the 12th European Conference on Eye Movements* (2003).

[2] Kliegl, R., Nuthmann, A. & Engbert, R. Tracking the mind during reading: the influence of past, present, and future words on fixation durations. *Journal of Experimental Psychology: General* **135**, 12–35 (2006). 16478314 10.1037/0096-3445.135.1.12

[3] Cop, U., Dirix, N., Drieghe, D. & Duyck, W. Presenting GECO: an eyetracking corpus of monolingual and bilingual sentence reading. *Behavior Research Methods* **49**, 602–615 (2017). 27193157 10.3758/s13428-016-0734-0

[4] Luke, S. G. & Christianson, K. The Provo corpus: a large eye-tracking corpus with predictability norms. *Behavior Research Methods* **50**, 826–833 (2018). 28523601 10.3758/s13428-017-0908-4

[5] Hollenstein, N. *et al*. ZuCo, a simultaneous EEG and eye-tracking resource for natural sentence reading. *Scientific Data* **5** (2018). 10.1038/sdata.2018.291 PMC6289117 30531985

[6] Ahn, S., Kelton, C., Balasubramanian, A. & Zelinsky, G. Towards predicting reading comprehension from gaze behavior. In *ACM Symposium on Eye Tracking Research and Applications*, 1–5 Association for Computing Machinery (2020).

[7] Berzak, Y. *et al*. CELER: a 365-participant corpus of eye movements in L1 and L2 English reading. *Open Mind* **6**, 41–50 (2022). 36439073 10.1162/opmi_a_00054 PMC9692049

[8] Siegelman, N. *et al*. Expanding horizons of cross-linguistic research on reading: the multilingual eye-movement corpus (MECO). *Behavior Research Methods* **54**, 2843–2863 (2022). 35112286 10.3758/s13428-021-01772-6 PMC8809631

[9] Demberg, V. & Keller, F. Data from eye-tracking corpora as evidence for theories of syntactic processing complexity. Cognition 109, 193-210 (2008). 10.1016/j.cognition.2008.07.008 18930455

[10] Smith, N. J. & Levy, R. The effect of word predictability on reading time is logarithmic. Cognition 128, 302-319 (2013). 10.1016/j.cognition.2013.02.013 PMC3709001 23747651

[11] Wilcox, E. G., Gauthier, J., Hu, J., Qian, P. & P. Levy, R. On the predictive power of neural language models for human real-time comprehension behavior. In *Proceedings of the 42nd Annual Meeting of the Cognitive Science Society*, 1707-1713 (2020).

[12] Eisape, T., Zaslavsky, N. & Levy, R. Cloze distillation: improving neural language models with human next-word prediction. In *Proceedings of the 24th Conference on Computational Natural Language Learning*, 609–619, (2020).

[13] Barrett, M. & Hollenstein, N. Sequence labelling and sequence classification with gaze: novel uses of eye-tracking data for natural language processing. *Language and Linguistics Compass* **14**, 1–16 (2020).

[14] Mathias, S., Kanojia, D., Mishra, A. & Bhattacharyya, P. A survey on using gaze behaviour for natural language processing. In *Proceedings of the 29th International Joint Conference on Artificial Intelligence and the 17th Pacific Rim International Conference on Artificial Intelligence*, 4907–4913 (2020).

[15] Deng, S. *et al*. Eyettention: an attention-based dual-sequence model for predicting human scanpaths during reading. *Proceedings of the ACM on Human-Computer Interaction* **7**, 1–24 (2023).

[16] Berzak, Y. *et al*. OneStop: a 360-participant English eye tracking dataset with different reading regimes. 10.17605/OSF.IO/2PRDQ (2025). 10.1038/s41597-025-06272-2 PMC12727756 41330931

[17] Berzak, Y., Malmaud, J. & Levy, R. STARC: structured annotations for reading comprehension. In *Proceedings of the 58th Annual Meeting of the Association for Computational Linguistics*, 5726–5735 (2020).

[18] Shubi, O. & Berzak, Y. Eye movements in information-seeking reading. In *Proceedings of the 45th Annual Meeting of the Cognitive Science Society*, 936–943 (2023).

[19] Shubi, O., Hadar, C. A. & Berzak, Y. Decoding reading goals from eye movements. In *Proceedings of the 63rd Annual Meeting of the Association for Computational Linguistics*, 5616–5637 (2025).

[20] Hadar, C. A., Shubi, O., Meiri, Y. & Berzak, Y. Decoding open-ended information seeking goals from eye movements in reading. *Preprint at* https://arxiv.org/abs/2505.02872 (2025).

[21] Meiri, Y. & Berzak, Y. Déjà vu: Eye movements in repeated reading. In *Proceedings of the 46th Annual Meeting of the Cognitive Science Society*, 5018-5024 (2024).

[22] Meiri, Y., Shubi, O., Hadar, C. A., Nitzav, A. K. & Berzak, Y. Déjà? decoding repeated reading from eye movements In *Proceedings of the 63rd Annual Meeting of the Association for Computational Linguistics*, 19460-19482 (2025).

[23] Gruteke Klein, K., Meiri, Y., Shubi, O. & Berzak, Y. The effect of surprisal on reading times in information seeking and repeated reading. In *Proceedings of the 28th Conference on Computational Natural Language Learning*, 219–230 (2024).

[24] Gruteke Klein, K., Frenkel, S., Shubi, O. & Berzak, Y. Readability formulas, systems and LLMs are poor predictors of reading ease. *Preprint at* https://arxiv.org/abs/2502.11150 (2025).

[25] Gruteke Klein, K., Shubi, O., Frenkel, S. & Berzak, Y. The effect of text simplification on reading fluency and reading comprehension in L1 English speakers. In *Proceedings of the 47th Annual Meeting of the Cognitive Science Society*, 742-749 (2025).

[26] Malmaud, J., Levy, R. & Berzak, Y. Bridging information-seeking human gaze and machine reading comprehension. In *Proceedings of the 24th Conference on Computational Natural Language Learning*, 142–152 (2020).

[27] Shubi, O., Meiri, Y., Hadar, C. A. & Berzak, Y. Fine-grained prediction of reading comprehension from eye movements. In *Proceedings of the 2024 Conference on Empirical Methods in Natural Language Processing*, 3372–3391 (2024).

[28] Vajjala, S. & Lučić, I. OneStopEnglish corpus: a new corpus for automatic readability assessment and text simplification. In *Proceedings of the Thirteenth Workshop on Innovative Use of NLP for Building Educational Applications*, 297–304 (2018).

[29] Rayner, K. & Liversedge, S. P. Linguistic and cognitive influences on eye movements during reading. In Liversedge, S., Gilchrist, I. & Everling, S. (eds.) *The Oxford Handbook of Eye Movements*, 751–766, Oxford University Press (2011).

[30] Clifton Jr, C. *et al*. Eye movements in reading and information processing: Keith Rayner’s 40 year legacy. *Journal of Memory and Language* **86**, 1–19 (2016).

[31] Honnibal, M., Montani, I., Van Landeghem, S. & Boyd, A. explosion/spaCy: industrial-strength natural language processing in Python, 10.5281/zenodo.1212303 (2020).

[32] Berzak, Y. *et al*. OneStop Ordinary Reading, 10.17605/OSF.IO/ZN9SQ (2025).

[33] Berzak, Y. *et al*. OneStop Information Seeking, 10.17605/OSF.IO/KPBGX (2025).

[34] Berzak, Y. *et al*. OneStop Repeated Reading, 10.17605/OSF.IO/4AY3T (2025).

[35] Berzak, Y. *et al*. OneStop Information Seeking in Repeated Reading, 10.17605/OSF.IO/6RA7T (2025).

[36] Berzak, Y. *et al*. OneStop All Regimes, 10.17605/OSF.IO/AZJ2G (2025).

[37] Berzak, Y. *et al*. OneStop Full, 10.17605/OSF.IO/Z7PYN (2025).

[38] Berzak, Y. *et al*. OneStop Raw Data, 10.17605/OSF.IO/6F2KM (2025).

[39] Berzak, Y. *et al*. OneStop Metadata, 10.17605/OSF.IO/JBD24 (2025).

[40] Rayner, K., Ashby, J., Pollatsek, A. & Reichle, E. D. The effects of frequency and predictability on eye fixations in reading: implications for the E-Z reader model. *Journal of Experimental Psychology: Human Perception and Performance* **30**, 720 (2004). 15301620 10.1037/0096-1523.30.4.720

[41] Kliegl, R., Grabner, E., Rolfs, M. & Engbert, R. Length, frequency, and predictability effects of words on eye movements in reading. *European Journal of Cognitive Psychology* **16**, 262–284 (2004).

[42] Rayner, K., Slattery, T. J., Drieghe, D. & Liversedge, S. P. Eye movements and word skipping during reading: effects of word length and predictability. *Journal of Experimental Psychology: Human Perception and Performance* **37**, 514 (2011). 21463086 10.1037/a0020990 PMC3543826

[43] Radford, A. *et al*. Language models are unsupervised multitask learners. *OpenAI blog*, https://cdn.openai.com/better-language-models/language_models_are_unsupervised_multitask_learners.pdf (2019).

[44] Speer, R., Chin, J., Lin, A., Jewett, S. & Nathan, L. Luminosoinsight/wordfreq, 10.5281/zenodo.1443582 (2018).

[45] Dryer, M. S. & Haspelmath, M. (eds.) The World Atlas of Language Structures Online (v2020.3), 10.5281/zenodo.7385533 (2013).

[46] Acheson, D. J., Wells, J. B. & MacDonald, M. C. New and updated tests of print exposure and reading abilities in college students. *Behavior Research Methods* **40**, 278–289 (2008). 18411551 10.3758/brm.40.1.278 PMC3022331

[47] Brysbaert, M. & New, B. Moving beyond Kučera and Francis: a critical evaluation of current word frequency norms and the introduction of a new and improved word frequency measure for American English. *Behavior Research Methods* **41**, 977–990 (2009). 19897807 10.3758/BRM.41.4.977

[48] De Marneffe, M.-C., Manning, C. D., Nivre, J. & Zeman, D. Universal dependencies. *Computational Linguistics* **47**, 255–308 (2021).

[49] Hollenstein, N., Troendle, M., Zhang, C. & Langer, N. ZuCo 2.0: a dataset of physiological recordings during natural reading and annotation. In *Proceedings of the 12th Language Resources and Evaluation Conference*, 138–146 (2020).

[50] Frank, S. L., Monsalve, I. F., Thompson, R. L. & Vigliocco, G. Reading time data for evaluating broad-coverage models of English sentence processing. *Behavior Research Methods* **45**, 1182–1190 (2013). 23404612 10.3758/s13428-012-0313-y

[51] Rayner, K. Eye movements in reading and information processing: 20 years of research. *Psychological Bulletin* **124**, 372 (1998). 9849112 10.1037/0033-2909.124.3.372

[52] Bezanson, J., Edelman, A., Karpinski, S. & Shah, V. B. Julia: a fresh approach to numerical computing. *SIAM Review* **59**, 65–98 (2017).

